# The Association Between BMI and Cardiorespiratory Functions Among Medical Students at Northern Border University

**DOI:** 10.7759/cureus.63191

**Published:** 2024-06-26

**Authors:** Mohamed M Abd El Mawgod, Hassan Mohammad, Zulfiqar A Abdulsattar, AbdulGhaffar Abdulrehman, Fahad A Almaradhi, Yousef M Alenzi, Abdulmohsen M Alanazi, Abdulrahman A Alanazi

**Affiliations:** 1 Family and Community Medicine, College of Medicine, Northern Border University, Arar, SAU; 2 Public Health and Community Medicine, Faculty of Medicine, Al-Azhar University, Assiut, EGY; 3 Anatomy, College of Medicine, Northern Border University, Arar, SAU; 4 Physiology, College of Medicine, Northern Border University, Arar, SAU; 5 Medicine, College of Medicine, Northern Border University, Arar, SAU

**Keywords:** medical students, mean arterial pressure, pulse pressure, tidal volume, respiratory rate, blood pressure, bmi

## Abstract

Background: Cardiorespiratory function is one of the key health indicators that promote good health. Knowing the correlation between body mass index (BMI) and cardiorespiratory functioning might assist in the creation of evidence-based therapies that focus on addressing difficulties associated with obesity.

Objective: To assess the correlation between BMI and cardiorespiratory functions among medical students at Northern Border University.

Materials and methods: A cross-sectional study was conducted among medical students at Northern Border University, Saudi Arabia. The blood pressure (BP), respiratory rate (RR), heart rate (HR), mean arterial pressure (MAP), pulse pressure (PP), and BMI of the students were measured.

Results: The mean age of the students was 17.1 ± 1.9 years. Nearly 40% of students were overweight or obese. Our study revealed a significant positive correlation between BMI and BP, RR, tidal volume (TV), and MAP.

Conclusions: The correlation analysis of our study revealed a significant positive correlation of BMI with BP, RR, TV, and MAP.

## Introduction

Overweight and obesity are abnormal or excessive fat buildups that pose a health concern [1]. They are measured as body mass index (BMI). Obesity is one of the world's major causes of early mortality [2]. The following formula can be used for BMI calculation: BMI = weight (kg)/height (m^2^). BMI is used by the WHO to categorize nutritional statuses. A BMI of less than 18.5 indicates underweight, a BMI of 18.5-24.9 indicates properly fed, and a BMI of more than 30 indicates obesity [3]. Reduced physical activity, trans fat consumption, co-morbidities, diabetes, hypertension, heart and endocrine problems, cancer, and other environmental factors negatively affect body weight and raise body mass index [4]. An individual's BMI plays a critical role in predicting potential future health issues, and maintaining a normal range is a reasonable goal for leading a healthy lifestyle [4].

Previous research has shown that, even in the absence of pulmonary diseases, obese individuals are frequently worn out from regular work [5]. In the general population, pulmonary function is a strong predictor of future illness and death [6]. Sustaining optimal lung function throughout adulthood is crucial for averting chronic respiratory disorders, which currently pose a significant global public health concern [7]. Obese people have been found to have aberrant pulmonary function parameters, such as lung volumes and respiratory efficiency [8]. Globally, BMI and blood pressure (BP) are both rising. According to epidemiological research, there is a positive linear relationship between the two [9]. A correlation between BMI and the risk of hypertension was shown in cohort research including medical students, highlighting the fact that even a little increase in weight during childhood might substantially increase the chance of getting hypertension in later life [10]. Furthermore, results from additional cohort research carried out in Israel highlighted that a rise of one unit in body mass index was linked to an elevated risk of hypertension [11]. These results demonstrate a strong and conclusive relationship between BMI and hypertension. The current study seeks to assess the associations between BMI and cardiorespiratory functions using a sample of medical students in Saudi Arabia.

## Materials and methods

Study setting and design

A cross-sectional study was conducted among medical students at Northern Border University, Saudi Arabia, during the period from October 1, 2023, to April 30, 2024.

Study tools

A structured questionnaire that included the following information was used: socio-demographic information, such as age, academic level, smoking habit, and living status, the practice of physical activity, the kind and frequency of physical exercise, and eating and sleeping habits variables, such as junk food consumption, and sleeping hours per day.

Using the height and weight measuring device, participants' body weight and height were recorded while they were dressed comfortably and without shoes. Pulmonary function tests were assessed using a portable spirometer. Tidal volume is the amount of air that moves in or out of the lungs during a single breath under normal, resting conditions. The normal value for tidal volume in adults typically ranges from 350 to 500 milliliters per breath. After being seated and having their nose clips fitted, the subjects were instructed to avoid flexion or extension. The number of breaths per minute was used to compute the respiratory rate.

Heart rate was measured using a heart rate monitor. BP was measured by an automated BP monitor. The mean arterial pressure (MAP) is the average pressure in a person's arteries during one cardiac cycle. It is a weighted average of systolic and diastolic BP and is an important indicator of tissue perfusion. The normal range for MAP is typically between 70 and 100 mmHg. MAP is calculated by the following formula: MAP = DP + 1/3 (SP - DP) or MAP = DP + 1/3 (PP). Where DP is the diastolic BP, SP is the systolic BP, and PP is the pulse pressure.

Pulse pressure (PP) refers to the difference between the systolic and diastolic BP readings. It reflects the force exerted by the heart when it contracts (systolic pressure) and the pressure in the arteries when the heart is at rest (diastolic pressure). Normal values for PP typically range from 30 to 40 mmHg, although they can vary depending on factors such as age, health status, and individual differences. PP is calculated as follows: PP = systolic blood pressure - diastolic blood pressure.

Sample size

The sample size was calculated using Epi Info program version 7.2.4.0 (Centers for Disease Control and Prevention, Atlanta, GA) at 80% power with a margin of error of 0.05, and the expected sample size was 195 participants.

Sampling methods

A convenient sampling method was used to conduct a survey through an interview. Every study participant provided informed consent, which was included at the beginning of the questionnaire.

Statistical analysis

Data analyses were done using SPSS version 20 (IBM SPSS Statistics for Windows, IBM Corp., Armonk, NY). The categorical variables are displayed as frequency and percentage, while the numerical data are presented as mean ± standard deviation. Correlation analysis was done by Pearson's correlation test. The P-value was considered significant at <0.05.

Inclusion criteria

Medical students from 1st year to 6th year who were willing to participate were included in the study.

Exclusion criteria

Non-medical students and students with complaints of cardiopulmonary diseases were excluded.

Ethical consideration

We obtained ethical approval from the Local Committee of Bioethics at Northern Border University (HAP-09-A-043) with decision number 95/23/H (dated: 12-10-2023). There was no risk to participants because the study was questionnaire-based, and students had complete control over whether to participate or not. Data privacy and confidentiality were assured.

## Results

Table 1 illustrates the demographic data of the studied subjects. A total of 195 participants were included in the study, their mean age was 17.1 ± 1.9 years, nearly 40% of them were overweight or obese, slightly more than one-fifth were smokers, and the majority were living with their families.

**Table 1:** Demographic data of the studied subjects.

Items	No. (195)	%
Age in years	Mean ± SD = 17.1 ± 1.9	
Academic grade		
1^st^ year	27	13.8
2^nd^ year	33	16.9
3^rd^ year	32	16.4
4^th^ year	33	16.9
5^th^ year	38	19.5
6^th^ year	38	19.5
Living status		
Dormitory	4	2.1
Rent	14	7.2
With family	177	90.8
Body mass index		
Underweight	11	5.6
Normal	109	55.9
Overweight	47	24.1
Obese	28	14.4
Smoking habits		
Yes	43	22.1
No	144	73.8
Ex-smoker	8	4.1

Table 2 describes the practice of physical activity among the studied participants. More than two-thirds of them performed physical activities. The most common physical activity among them was going to the gym (41%, 55). Slightly less than 40% of them spent more than three times a week on physical activity, and approximately a quarter of them spent more than one hour on physical activity.

**Table 2:** Physical activity status of the studied participants. * Number of subjects practicing physical activity = 134.

Items	No. (195)	%
Practice of physical activity		
Yes	134	68.7
No	61	31.3
Types of physical activity*		
Walking	37	27.6
Gym	55	41
Football	39	29.1
Other sports	3	2.2
How many times weekly?		
One time	20	14.9
Two times	36	26.9
Three times	26	19.4
More than three times	52	38.8
Time spent in physical activity		
Less than 15 min	8	6.0
15-30 min	24	17.9
30-45 min	29	21.6
45-60 min	40	29.9
More than one hour	33	24.6

Table 3 illustrates the food and sleep habits of the studied participants. More than half of them eat junk food, slightly less than 60% eat junk food daily, and surprisingly, around 40% sleep less than eight hours daily. The habit of eating junk food is associated with an increase in overweight and obesity.

**Table 3:** Food and sleep habits of the studied participants. * Number of participants eating junk food = 109.

Items	No. (195)	%
Eating junk food		
Yes	109	55.9
No	24	12.3
Sometimes	62	31.8
How many times do you eat junk food?^*^		
Daily	62	56.9
Two times weekly	14	12.8
Three times weekly	18	16.5
More than three times weekly	15	13.8
No. of sleeping hours per day		
5	8	4.1
6	26	13.3
7	42	21.5
8	60	30.8
9	18	9.2
10	34	17.4
12	7	3.6

Table 4 describes the correlation (r) of BMI with heart rate, pulse pressure, age, and diastolic blood pressure. The study showed an insignificant negative correlation between BMI and heart rate (P-value > 0.05). Additionally, there was a negligible positive correlation between BMI, pulse pressure, age, and diastolic blood pressure (P-value > 0.05) (Table 5).

**Table 4:** Correlation between BMI with heart rate, pulse pressure, age, and diastolic blood pressure. P-value = Pearson's correlation test; r = correlation coefficient.

Items	r	P-value
Heart rate	-0.03	0.6
Pulse pressure	0.13	0.06
Age	0.03	0.6
Diastolic blood pressure	0.11	0.12

**Table 5:** Correlation between BMI and tidal volume, respiratory rate, mean arterial pressure, and systolic blood pressure. P-value = Pearson's correlation test; r = correlation coefficient.

Items	r	P-value
Tidal volume	0.44	0.000
Respiratory rate	0.17	0.017
Mean arterial pressure	0.14	0.042
Systolic blood pressure	0.16	0.02

## Discussion

The results of the current survey indicate a statistically positive correlation between MAP and BMI. In line with a UK study that found a positive correlation between BMI and MAP [12]. Additionally, an Indian study among young adults stated that there was a significant correlation between BMI and MAP [13]. In the same country, research found that a significant decline in BMI and MAP was observed in the physically active population as compared to the sedentary population [14]. A study found the intricate relationships that existed between fat distribution, body composition, circadian systolic blood pressure, diastolic blood pressure, and MAP profiles of obese individuals [15].

Regarding the correlation between BMI and PP, the study reveals a positive correlation, but it is not statistically significant (P-value = 0.06). A multicenter cross-sectional study in China found a linear positive correlation between BMI and PP [16]. Research in Uganda showed that there was a significant difference in PP among the BMI categories (P < 0.01) [17]. A study in China found that brachial-ankle pulse wave velocity showed a positive linear association with PP (r = 0.53, P < 0.01) [18].

Our study reveals an insignificant positive correlation between BMI and age (P-value = 0.6). An Iranian study showed a positive correlation between age and BMI [19].

Regarding BMI and tidal volume, our research finds a significant positive correlation between them. In agreement with similar studies conducted in Saudi Arabia [20], India [21], and the United States [22], they cited a positive association between BMI and tidal volume. In contrast, Srinivas et al. [23] and Chen et al. in Canada [24] found a negative association between BMI and pulmonary function among overweight and obese people.

Our research shows a negative, non-significant correlation between BMI and heart rate (P-value = 0.6). In contrast, a Pakistani study showed a significant positive correlation between BMI and heart rate [25].

Current research illustrates a positive correlation between BMI and systolic and diastolic blood pressure, which is in agreement with similar studies conducted in Germany (P < 0.001) [26], Indonesia (P < 0.000) [27], and India (P < 0.001) [28,29]. Turkey (P < 0.000 and P < 0.01) [30,31], Pakistan (P < 0.004) [32], Brazil (P < 0.001) [33], and China (P < 0.05) [34] showed a positive correlation between BMI and BP.

Limitations of the study

This is a descriptive study, so we cannot determine the cause-effect relationship. We are dealing with a convenient sample, so the results cannot be generalized.

## Conclusions

Our study showed a significant positive correlation between BMI and systolic blood pressure, RR, and MAP among the medical students at Northern Border University, Saudi Arabia. It appears that gaining weight is a significant risk factor for the development of many diseases like hypertension, diabetes mellitus, and coronary artery disease. Promoting calorie restriction, physical exercise, and weight loss will help reduce obesity and the associated burden of cardiovascular disease worldwide. This research gives motivation for the early prevention of obesity among youth.
